# (Cost-)effectiveness of focal therapy versus radical therapy (standard of care) in the treatment of men with intermediate-risk prostate cancer: study protocol for the ENFORCE focal randomised controlled trial

**DOI:** 10.1136/bmjopen-2026-117276

**Published:** 2026-07-08

**Authors:** Lauren P W te Molder, J P Michiel Sedelaar, Joyce G R Bomers, Martijn F Boomsma, Tjard D de Haan, Harm H E van Melick, Martijn R Meijerink, Harrie P Beerlage, Olav van Aubel, Eelco R Collette, Janneke P C Grutters, Gerjon Hannink, Maroeska M Rovers, Jurgen J Fütterer, ENFORCE Focal consortium, Lauren P W te Molder

**Affiliations:** 1 Department of Medical Imaging, Radboudumc, Nijmegen, The Netherlands; 2 Department of Urology, Radboudumc, Nijmegen, The Netherlands; 3 Department of Radiology, Isala Hospital, Zwolle, The Netherlands; 4 Department of Urology, Isala Hospital, Zwolle, The Netherlands; 5 Department of Urology, St Antonius Ziekenhuis, Nieuwegein, The Netherlands; 6 Department of Urology, St Antonius Hospital, Utrecht, The Netherlands; 7 Department of Radiology and Nuclear Medicine, Amsterdam UMC, Amsterdam, The Netherlands; 8 Department of Urology, Amsterdam UMC Location VUmc, Amsterdam, The Netherlands; 9 Urologist, HIFU Clinic, Etten-Leur, The Netherlands; 10 Department of Urology, Bravis ziekenhuis, Bergen op Zoom, The Netherlands; 11 Department of IQ Health, Radboudumc, Nijmegen, The Netherlands

**Keywords:** Quality of Life, Prostate, Prostatic Neoplasms

## Abstract

**Background:**

Intermediate-risk prostate cancer (PCa) is currently managed with radical whole-gland therapies, such as radical prostatectomy (RP) or radiotherapy (RT). While effective, these treatments can significantly impact quality of life (QoL), particularly in relation to urinary incontinence and erectile dysfunction. Focal therapy poses a promising alternative for whole-gland treatment in this specific patient population; however, current evidence has primarily been limited to phase I and II studies. Robust prospective evidence is needed to determine whether focal therapy can be considered a safe alternative for management of intermediate-risk PCa. The objective of this randomised controlled trial (RCT) is to evaluate the oncological effectiveness, QoL and cost-effectiveness of focal therapy compared with radical treatment as standard of care.

**Methods:**

This ENFORCE focal study is designed as a multicentre RCT with six participating centres that perform focal therapy in the Netherlands. Patients are included after receiving informed consent and are thereafter 1:1 randomised between focal therapy, consisting of high-intensity focused ultrasound, irreversible electroporation or transurethral ultrasound ablation and radical treatment, consisting of RT or RP. A total of 356 patients will be enrolled. This study has two co-primary endpoints, oncological effectiveness at 36 months (non-inferiority) and QoL at 12 months (superiority) both of which must be met to demonstrate overall trial success. Follow-up will be at least 3 years, with a maximum of 5 years to gain information regarding long-term outcomes. The set follow-up visits are 6 weeks, 3 months, 6 months, 12 months and yearly thereafter. At the follow-up visits, clinical data will be collected, and questionnaires regarding QoL and costs will be administered. Furthermore, at 9 months and 18 months, prostate-specific antigen will be monitored in alignment with European guidelines on follow-up after RP and RT. Primary analyses will be conducted using intention-to-treat analyses.

**Ethics and dissemination:**

Ethical approval has been obtained from the Medical Research Ethical Committee Oost-Nederland (NL83913.091.23, primary approval in December 2023; last approved version 10 July 2025). The results will be submitted for publication in peer-reviewed medical journals, and data will be accessible.

**Trial registration:**

NCT06223295.

**STRENGTHS AND LIMITATIONS OF THIS STUDY**

- Comparison between the groups of therapies, rather than a one-to-one comparison of individual therapies.
- No differences have been observed between different focal therapies, but these techniques have not been directly compared within a single study.
- A difference in follow-up protocols between the treatment arms.
- Quality of life will be assessed using patient-reported questionnaires, which may be subject to bias.

## Introduction

 In current clinical practice, intermediate-risk prostate cancer (PCa) is typically managed using radical whole-gland treatment, such as robot-assisted radical prostatectomy (RP) or a form of radiotherapy (RT). These treatments are known to be associated with complications, mainly consisting of incontinence and erectile dysfunction, affecting 21% and 70% of patients with RP, respectively.^1^ For patients with RT, urinary complaints, such as frequency, urgency, urethral stricture or incontinence, are observed in up to 27% of the patients, erectile dysfunction in up to 29% and gastrointestinal toxicity in up to 11%.^2^ These side-effects from RT and RP can substantially impact quality of life (QoL) of patients.

PCa is a heterogenous disease, with a risk classification ranging from low to high risk, requiring different treatment approaches. While radical treatment for high-risk disease results in favourable oncological outcomes,^3^ evidence shows that active surveillance (AS) is a safe approach for low-risk PCa without increasing the risk of unfavourable outcomes.^4^ AS is close monitoring, using prostate-specific antigen (PSA), MRI and biopsies, of PCa without immediate treatment. Although intermediate-risk PCa often requires treatment, it is debatable if patients with less aggressive tumours than high-risk patients also require a radical whole-gland treatment.^5^

Patients are becoming more assertive, increasingly informed about available treatment options and more actively involved in making decisions about their healthcare, the so-called shared decision making. Furthermore, patients seem to be willing to sacrifice some survival to improve their QoL.^6 7^ In this context, focal therapy, which treats only the tumour-bearing portion of the prostate, may be a valuable treatment option for patients with intermediate-risk PCa. Over the past decade, research into focal therapy for PCa has explored different energy sources, such as high-intensity focused ultrasound (HIFU), irreversible electroporation (IRE), cryoablation, focal laser ablation (FLA) and photodynamic therapy (PDT).^7^ These different types of energy transmission all seem to be feasible focal treatments.^8^ All these focal therapies are aimed at patients with low- and intermediate-risk PCa. The concept of focal therapy is founded on the premise of preserving QoL while maintaining oncological control. The rationale for this concept is based on the index lesion theory.^9^ The index lesion theory holds that PCa is often multi-focal, with one index lesion being the main determining factor of tumour behaviour, aggressiveness and disease progression. Other lesions are often clinically insignificant and contribute not at all or in a much smaller portion to disease progression over a 10–20-year period.^10^ Focal therapy targets the ablation of the MRI-visible, biopsy-confirmed index lesion along with an appropriate safety margin, aiming to achieve oncological outcomes comparable to those of radical treatments in patients with low- or intermediate-risk PCa.^11 12^ Furthermore, treating only part of the gland is thought to preserve most of the function, potentially leading to a better QoL compared with whole-gland treatment. To achieve this precise treatment, it is necessary to use peri-operative image guidance, typically through MRI or ultrasound (US). Focal therapy has the potential to delay or even eliminate the need for radical treatment by slowing disease progression. Currently, most patients will undergo an AS regime after focal therapy, including PSA measures, MRI and possibly prostate biopsy. In the event of recurrence or the appearance of a new lesion, patients can still undergo radical treatment or receive additional focal therapy.^13^

Despite current research into focal therapies for PCa,^7^ there is still a lack of high-quality, Idea, Development, Exploration, Assessment, Long-term (IDEAL) stage 3 evidence, as most existing studies are limited to IDEAL stages 1, 2a and 2b. These previous studies often have considerable heterogeneity in study design, patient populations and ablative protocols, making it difficult to compare the efficacy of different ablation modalities.^8 14^ Furthermore, current evidence on the cost-effectiveness of focal ablation techniques is insufficient to inform guidance regarding their routine clinical use.^15^ This underlines the need for a multicentre randomised controlled trial (RCT) to determine the role of focal therapies in the treatment of intermediate-risk PCa. Currently, several RCTs are underway, often comparing one focal therapy modality with one type of standard of care, for example, RP, such as the CAPTAIN trial (trial registration: NCT05027477), the PRIS trial^16^ and the PART trial (trial registration: ISRCTN17249875). In contrast, our ENFORCE focal study adopts a broader approach by comparing multiple focal therapy modalities, consisting of HIFU, transurethral ultrasound ablation (TULSA) and IRE, against various radical standard-of-care treatments. This design distinguishes ENFORCE focal from other RCTs, producing more generalisable results helping real-world decision making in daily practice.

Our RCT aims to study the (cost-)effectiveness of focal therapy compared with radical whole-gland treatment in men with intermediate, unilateral PCa in terms of oncological effectiveness and QoL.

## Methods

### Design

ENFORCE focal is a pragmatic multicentre, RCT comparing the (cost-)effectiveness of focal therapy to radical whole-gland treatment, that is, RP or RT, in patients with intermediate-risk PCa. Patient recruitment will be performed in outbound patient clinics in six Dutch centres and their networks. Each participating centre performs a focal ablation technique and radial whole-gland treatment. Focal therapies applied in the study are TULSA, HIFU and IRE; each centre performs one of the focal therapies. Radical treatment will be performed as per normal routing. This means that RP will be performed by the urologist in the participating centres or in their collaborative network, and patients choosing RT will be referred to RT centres. Within the trial, one of the participating centres collaborates with a dedicated HIFU clinic where the focal treatment is carried out. Follow-up will be at least 3 years, with a maximum of 5 years to gain information regarding long-term outcomes. The set follow-up visits are 6 weeks, 3 months, 6 months, 12 months and yearly thereafter. At the follow-up visits clinical data will be collected, and questionnaires regarding QoL and costs will be administered. Furthermore, at 9 months and 18 months PSA will be monitored in both groups in alignment with European guidelines on follow-up after RP and RT. For the focal therapy arm, at 12 months, an MRI and prostate biopsies will be performed. In the years thereafter, a prostate MRI will be performed yearly with additional biopsies on indication, such as rising PSA levels over time, suspicious MR findings in the treated zone or newly identified lesions outside the treated area.

### Outcome measures

#### Primary endpoints

The co-primary outcomes are oncological effectiveness (non-inferiority) and QoL (superiority). Oncological effectiveness is defined as treatment failure, that is, pathologically proven clinically significant (meaning Gleason 7 or higher) recurrent disease and/or the medical indication for retreatment with salvage treatment (RP, RT or systemic treatment) in the focal therapy group, and as a biochemical recurrence (defined as at least two PSA values that are 0.2 ng/mL or higher in the RP group and according to the Phoenix criteria (increase in PSA of≥2 ng/mL above nadir) in the RT group) and/or salvage local or systemic treatment in the radical whole-gland treatment group. The primary endpoint for oncological effectiveness is set at 36 months of follow-up. QoL will be measured using the functional assessment of cancer therapy prostate cancer (FACT-P) overall score at 12 months. The FACT-P is a disease-specific questionnaire for PCa that evaluates an overall score to assess the QoL as well as per domain. The questionnaire contains five domains, including 27 questions in total and prostate-specific questions consisting of 12 questions. The domains will be assessed as secondary outcome measures.

#### Secondary endpoints

Secondary endpoints include metastasis-free survival, generic and disease-specific patient-reported health-related QoL using validated questionnaires, oncologic safety, disease progression, disease-specific mortality, all-cause mortality, adverse events, operating time, hospital care stay, pathology results, resources use and productivity loss using validated questionnaires and cost-effectiveness. The questionnaires will be completed online using a secure web-based module at baseline and at specific follow-up intervals, that is, 6 weeks, 3 months, 6 months, 12 months and annually thereafter for up to 60 months.

For the patient-reported health-related QoL outcomes, patients will use a set of six validated questionnaires. QoL is measured at baseline and at set points during follow-up.

For the other secondary endpoints the following questionnaires will be used: European Organisation for Research and Treatment of Cancer QoL questionnaire prostate 25 (EORTC QLQ PR-25), international consultation on incontinence questionnaire (ICIQ), international prostate symptom score (IPSS), sexual health inventory measurement international index of erectile dysfunction (SHIM IIEFF-5), EuroQoL 5 dimensions five-level version (EQ-5D-5L). The EORTC QLQ PR-25 is a questionnaire to assess sexual problems and functioning, micturition, bowel symptoms and hormonal treatment side effects. This questionnaire contains 25 questions regarding those aspects. The ICIQ is used to assess urinary function and its impact on QoL and contains four questions. IPSS is a questionnaire for assessment of the degree of lower urinary tract symptoms and QoL. This score consists of seven questions and an overall score on quality of miction. The SHIM IIEF-5 used is the 5-item version of the IIEF. This questionnaire assesses erectile function and contains five questions. The EQ-5D-5L is a questionnaire that assesses general health in five dimensions. These dimensions are mobility, self-care, usual activities, pain/discomfort and anxiety/depression. Further, a visual analogue scale (VAS) is used to provide a quantitative measure of the perception of overall health of a participant.

Two questionnaires will additionally be used regarding cost-effectiveness analyses. The iMTA productivity cost questionnaire (iPCQ) is used to assess productivity loss in terms of absence from work due to illness. The iMTA medical costs questionnaire (iMCQ) is used to determine healthcare consumption, including hospital visits and domestic help.

In the secondary endpoints, clinical outcomes are included, such as operating time and hospital care stay. Operating time will be registered for focal therapies as the time between the start of imaging and the last performed imaging and for RP as the time from the start of incision until closing. For RT, we will register the type of radiation given. Further, data will be collected on treatment-related complications occurring during or after the procedure. During follow-up oncologic safety, in terms of disease progression, metastasis-free survival, disease-specific mortality and all-cause mortality, will be assessed using PSA, MRI and biopsies, as well as data from a national registry. Pathology results from surgery and biopsies will be collected. In addition to the questionnaires for cost-effectiveness analysis, hospital resource use will be recorded.

### Sample size

The hypothesis of this trial is that focal therapy is at least as effective as radical whole-gland treatment on oncological effectiveness while improving QoL. Oncological effectiveness and QoL are co-primary outcomes in this study. The non-inferiority margin was set at 15%, based on an anticipated 5% difference in oncological effectiveness and an additional 10% margin. The 5% difference was estimated from published event rates and the clinical experience of participating urologists, while the additional 10% margin was defined and agreed upon by both patients and relevant medical societies for the focal therapy group. To detect non-inferiority (80% power, one-sided alpha 5%), 356 participants are required. A drop-out rate of 5% is anticipated, allowing inclusion up to a total of 187 participants per group. To detect improvement (superiority) in QoL on the FACT-P overall score (90% power, one-sided alpha 5%), with a minimally clinically important difference (MCID) of 8 and a SD of 20,^17^ a total of 132 patients are required per arm.

Trial success is defined as achieving statistical significance for both co-primary endpoints simultaneously. To ensure adequate probability of joint success, the sample size was calculated to achieve an overall joint power of 80%.

### Patient characteristics

Patients aged 40 years and older with localised unilateral dominant PCa, a PSA≤20 ng/mL and normally eligible for general anaesthesia will be included in this study. All inclusion and exclusion criteria are listed in table 1. Patients will be included after being diagnosed with intermediate-risk PCa and having completed the diagnostic work-up according to national guidelines. At a minimum, all patients will have undergone clinical examination, PSA testing, prostate MRI and prostate biopsies. Additional staging procedures are not part of standard staging for this risk category in the Netherlands.

**Table 1:** Inclusion and exclusion criteria. To be able to participate in this study, patients must meet all inclusion criteria and cannot meet any of the exclusion criteria

Inclusion criteria	Exclusion criteria
Men, age of 40 years or over, with unilateral clinically significant intermediate-risk PCa or dominant unilateral clinically significant intermediate-risk and small contralateral low-risk (ISUP) one disease	Unfit for general anaesthesia or radical surgery
Gleason score of 7 (3+4 or 4+3; ISUP grade 2/3)	Low-volume low-risk disease (≤4 mm Gleason score of≤6/ISUP grade 1)
PSA level of≤20 ng/mL	High-risk disease (Gleason score of≥8/ISUP grade>3)
Clinical stage≤T2b disease	Clinical cT2c or T3 disease (extracapsular PCa)
Life expectancy of≥10 years	Men who have received previous active therapy for PCa
Men with a prostate size≤5 cm in sagittal length or≤6 cm in axial length	Men with evidence of extraprostatic disease
Fit, eligible and normally destined for radical surgery or radiotherapy	Men with an inability to tolerate transrectal ultrasound
No concomitant cancer	Cardiac pacemaker
No previous treatment of their prostate	Metal implants/stent in the urethra or prostate
An understanding of the Dutch language sufficient to receive written and verbal information about the trial, its consent and the study questionnaires	ASA≥4
	Prostatic calcifications/cysts that interfere with effective delivery of TULSA/HIFU based on MR
	Men with renal impairment and a glomerular filtration rate (GFR) of<30 mL/minute/1.73m2
	Unable to give consent to participate in the trial, as judged by the attending clinicians

GFR, glomerular filtration rate; HIFU, high-intensity focused ultrasound; PCa, prostate cancer; PSA, prostate-specific antigen; TULSA, transurethral ultrasound ablation.

### Randomisation

Eligible participants who provide informed consent will be randomised in a 1:1 ratio to focal therapy or radical whole-gland treatment using a computer-generated allocation sequence. Randomisation will be stratified by centre, Gleason score (3+4 or 4+3) and age group to ensure balanced distribution of participants across treatment arms within each stratum.

Random block sizes will be used within each stratum to maintain allocation concealment. The allocation sequence will be implemented via a centralised web-based system to maintain blinding of investigators and study personnel involved in enrolment.

### Interventions

The therapies within the intervention group are HIFU, TULSA and IRE. If a patient is assigned to focal therapy, the treatment a patient receives is based on the centre where the inclusion took place. This means that a patient is not able to choose what kind of focal therapy will be received in case of randomisation for focal therapy. The control group consists of RP or RT. If a patient is allocated to radical treatment, the patient will receive treatment as per current clinical practice in the Netherlands according to the Dutch guidelines. For RP, different types of approaches are possible, including nerve-sparing and non-nerve-sparing options, depending on tumour characteristics and anatomical considerations. Patients receiving RT will be treated as per national guidelines. Therapies administered can include RT with hypofractionation (28 fractions with 2.5 Gy, total dose of 70 Gy or 20 fractions with 3.1 Gy, total dose of 62 Gy) and RT with ultrahypofractionation (5 fractions with 7.25 Gy, total dose of 36.25 Gy). All RT regimens provide bio-equivalent doses similar to conventional RT consisting of 35 fractions with 2 Gy. According to the current national guidelines, the addition of androgen deprivation therapy (ADT) is not recommended for patients with intermediate-risk PCa with a PSA level of 20 ng/mL or lower. Patients eligible for both RP and RT are provided with evidence-based information, including a decision aid, to support shared decision making. The final treatment choice is made jointly by the patient and the urologist.

### Recruitment

Patients will be recruited from the six participating centres and their regional networks. Two of the including centres will perform TULSA, two will perform IRE and two centres will perform HIFU. One of the centres that performs HIFU collaborates with the including hospital in which the usual care arm will be performed.

#### Consent

All potential participants receive verbal and written information. After 7 days a member of the research team has telephone contact with the potential participant. If the patient is still interested in participating in the study, the informed consent papers (onlinesupplemental material 1  2 - (‘Subject Information Participation_ENFORCE’ and ‘Informed consent form_ENFORCE’) are signed in duplicate by the patient. After receipt of the signed papers, these are also signed by a research team member, which makes the enrolment in the study official.

### Analysis

Descriptive statistics will be used to summarise the data. For the oncological effectiveness, a one-sided 95% upper confidence bound will be created for the percentage difference in pathologically proven recurrent disease and/or (indication for) re-treatment (salvage treatment). If this bound is less than the non-inferiority margin of 15%, focal therapy will be declared to be non-inferior to usual care, that is, RP/RT. For the QoL, outcome differences between the two groups will be calculated with 95% CIs. All data will be analysed according to the intention-to-treat principle, including all randomised participants in the groups to which they were originally assigned, regardless of protocol deviations or loss to follow-up. In addition, to test robustness of the results, per-protocol and as-treated analysis will be performed regarding oncological effectiveness and QoL.

Furthermore, for secondary QoL outcomes, individual item responses will be used to derive the proportion of men reporting a moderate or big problem (or equivalent).

Metastasis-free survival, disease progression, disease-specific mortality and all-cause mortality will be presented with Kaplan-Meier curves. Procedure time and hospital care stay will be summarised using descriptive statistics. Treatment-related complications will be recorded according to the Clavien-Dindo classification. An overall risk difference for the overall complication rate and a 95% CI, and a risk difference per complication and their corresponding 95% CIs will be calculated. Pathology results will be summarised using descriptive statistics.

Cost-effectiveness will be performed from a societal perspective as per Dutch guidelines for economic evaluation.^18^ Costs will be calculated according to the Dutch guideline for costing research. Average costs will be calculated in both groups and differences will be calculated inclusive of 95% CIs. Effectiveness will be measured in terms of quality-adjusted life years (QALYs). These will be based on the utility scores as measured with the EQ-5D-5L, using the area under the curve method. Incremental cost-effectiveness ratios will be calculated by dividing estimated differences in costs by differences in QALYs. Uncertainty will be addressed by means of non-parametric bootstrapping and, where relevant, one-way sensitivity analyses.

A detailed Statistical Analysis Plan will be developed and finalised prior to database lock, outlining all pre-specified analyses and methods for handling missing data.

### Patient and public involvement statement

Patient organisations regarding PCa were involved in the design, especially in defining the outcomes, that is, non-inferiority margin. Patients or the public were not involved in the recruitment of participants. However, within this study patients are being recruited for participation. Results will be shared with participants and patient organisations; after dissemination of the results, patient organisations can also further share the outcomes of this study through websites and magazines.

## Ethics and dissemination

###  Ethical approval

The protocol, informed consent form, participant information and amendments are reviewed and approved by the sponsor (the National Health Care Institute and ZonMw). Ethical approval has been obtained from the local accredited Medical Research Ethics Committee (NL83913.091.23, primary approval in December 2023; last approved version 10 July 2025), MREC Oost-Nederland. The approval is valid for all participating centres. Besides the ethical approval for the study, local feasibility was obtained in all participating centres.

### Dissemination

Manuscripts with the results will be published in peer-reviewed medical journals. The data will be stored in a dedicated and secure workspace of the Digital Research Environment (anDREa). Access to the data within the anDREa is available everywhere. The metadata will be published at the certified DANS EASY public archive. In our publications, we will include links to the datasets used for publication.

## Discussion

Currently, the standard of care in intermediate-risk PCa includes RP and RT.^3^ However, there is growing interest among patients and physicians in alternative, organ-sparing approaches such as focal therapy. Our study, ENFORCE focal, is designed to generate high-quality evidence through an RCT comparing focal therapy to the current standard of care. The key strength of this design is the comparison between the groups of therapies rather than a one-to-one comparison of individual therapies, which distinguishes this study from other RCTs on focal therapy.^19^ Therapies in the focal therapy group are selected based on the available clinical experience in the Netherlands. Prostate MRIs are routinely performed in Dutch hospitals, so eligible patients can be referred by non-participating centres to the study sites. We are confident that recruitment of the required number of patients to evaluate (cost-)effectiveness and QoL can be achieved within the planned 2.5-year timeframe. The geographical distribution of including centres will ensure good national coverage within the Netherlands.

Our study is recognised as important for potential treatment options for patients with intermediate-risk PCa and is funded by the programme ‘Potentially Promising Care’ (Dutch: Veelbelovende Zorg) by the National Health Care Institute and ZonMw (ZorgOnderzoek Nederland Medische Wetenschappen). This programme is aimed at funding clinical studies that collect evidence on promising but relatively costly treatments. One of the key objectives of this study under this grant is to contribute to existing evidence of focal therapy and to contribute to the decision-making whether these treatment options will be reimbursed and included in the standard care for intermediate-risk PCa.

We expect several challenges in executing our study. First, in previous studies no differences have been observed between the different focal therapies,^8 14^ but these techniques have not been directly compared within a single study. We aim to gain more insights into potential existing differences between the different modalities. For this aim, we will perform exploratory analyses for the focal therapies versus the radical treatments. Adjustment for centre will not be possible in these analyses since the type of focal therapy is centre-dependent; also the offered standard of care modalities differ between the centres. Furthermore, the different centres may have different perioperative protocols or complications.

Second, another complexity in this study is the difference in follow-up protocols between the treatment arms. In the standard of care arm, PSA is used as the primary follow-up marker. After RP the PSA level is expected to become undetectable within 2 months.^20^ PSA levels in RT drop more slowly compared with RP. No optimal cut-off value has been determined, but in current clinical practice a PSA<0.5 ng/mL is set as a favourable outcome after RT.^21^ However, after focal therapy, PSA monitoring becomes less reliable since the untreated part of the prostate continues to function and produce PSA as before. This necessitates a different follow-up approach for patients in the focal therapy arm. In this trial, we will perform MRI and biopsies as an additional part of the follow-up after focal therapy, as MRI has proven effective in detecting new or recurrent lesions in the prostate. Recently, scoring systems have been developed to evaluate post-focal treatment MRIs, in which the likelihood of recurrent disease is scored.^22 23^ These systems offer guidance on subsequent steps, ranging from continued observation to additional diagnostic procedures such as biopsies. We will implement such a scoring system (PI-FAB scoring system) alongside standardised biopsies at 1-year follow-up to confirm the presence or absence of a tumour after focal treatment. This approach allows us to validate the scoring system by comparing its outcomes with biopsy results.

Third, QoL will be assessed using patient-reported questionnaires, which may be subject to bias such as recall bias and response bias. These questionnaires are, however, validated, and the use of these questionnaires aims to reduce these biases.

In conclusion, this ongoing RCT aims to contribute to the evidence needed to define the role of focal therapy in the treatment of intermediate-risk PCa, despite persistent methodological and clinical challenges.

### Trial status

Recruitment started in January 2024 with the first patient being included in February and is currently ongoing in six centres. A total of 110 patients are enrolled in October 2025, and we expect to need 2.5 years in total for the inclusion of 356 patients. A 1-year follow-up regarding QoL is expected for the whole group at the end of 2027. For the oncological effectiveness, the 3-year follow-up is expected at the end of 2029.

### Focal therapies

#### Transurethral ultrasound ablation

TULSA is a treatment in which a prostate tumour is treated with US under MR guidance. For this procedure, patients receive general anaesthesia. Afterwards, a patient will be positioned on the MR table. The transurethral device (UA) is inserted into the prostate, and the rectal cooling device is placed inside the rectum. The UA has 10 US elements which can individually provide energy. After positioning the patient and device, MR imaging is performed. Treatment planning is performed by the clinician and the TULSA system. Since the TULSA will be a focal approach, a partial gland ablation will be performed. After the procedure, a post-ablation scan will be performed to determine the treated area. If the treatment has been performed to the satisfaction of the clinician, the device will be removed and a catheter will be placed. Patients will be admitted for one night. They will be discharged from the hospital if in good clinical condition the day after.

#### Irreversible electroporation

IRE is an US-guided treatment. In this treatment, patients will receive general anaesthesia and muscle relaxation. After induction, patients will be positioned in the lithotomy position. Needles are then transperineally placed with the use of a transrectal US. By using a grid, the needles will be placed around the tumour. After placement, pulsatile high-voltage electric currents will be run between the needles in pulses. These pulses generate an electric field that permeabilises the cell membranes within the treatment zone and thus includes the tumour cells. The formation of irreversible nanopores in the cellular membrane leads to a disruption of the cell membrane, resulting in cell death. Periprocedural imaging with US and the planning software of the IRE help in ensuring complete coverage of the area of interest. A catheter will be placed during the treatment, and patients will be discharged from the hospital the same day or after 1 day if in good clinical condition.

#### High-intensity focused ultrasound

HIFU is an US-guided treatment while using HIFU to treat a prostate tumour. Patients receive spinal anaesthesia with sedation for this treatment. After the anaesthesia is performed, a catheter is placed, and patients will be positioned in the right lateral decubitus position. After positioning, the endorectal device is inserted. The diagnostic MRI is fused with real-time US imaging for treatment planning. After the fusion, the HIFU device performs the treatment planning. After planning, the ablation will be performed using real-time US guidance. This is also used to determine the treated area is sufficient for the treatment of the lesion of interest. If patients are in good clinical condition, they will be discharged the same day as the treatment takes place.

## Supplementary material

10.1136/bmjopen-2026-117276 online supplemental file 1

10.1136/bmjopen-2026-117276 online supplemental file 2
