# Supplementary material for: (Cost-)effectiveness of focal therapy versus radical therapy (standard of care) in the treatment of men with intermediate-risk prostate cancer: study protocol for the ENFORCE focal randomised controlled trial
Source: BMJ Open. 2026 Jul 8;16(7):e117276. doi: 10.1136/bmjopen-2026-117276 (PMC13347909; doi:10.1136/bmjopen-2026-117276)
Supplement: online supplemental file 1 [file bmjopen-16-7-s001.docx]

*Disclaimer: this document is an English translation of the original Dutch informed consent form for publication purposes. Only the Dutch version was approved by the Medical Ethics Review Committee and used during participant enrollment.*

**Subject Information Participation in Medical Scientific Research**

**Effectiveness of focal therapy in men with prostate cancer (ENFORCE)**

**Introduction**

Dear Sir,

With this information letter, we would like to ask whether you would like to participate in medical scientific research. Participation is voluntary. You are receiving this letter because you have prostate cancer and may be eligible to participate in this study.

In this document, you can read what the study involves, what it means for you, and what the advantages and disadvantages are. It is a lot of information. Please read the information carefully and decide whether you wish to participate. If you would like to participate, you can complete the form included in Appendix E.

**Ask your questions**

You can make your decision using the information in this information letter. In addition, we recommend that you do the following:

- Ask questions to the researcher who is providing you with this information.
- Discuss this study with your partner, family, or friends.
- Ask questions to the independent expert, Dr. F. Martens (contact details see Appendix A).
- Read the information at [www.rijksoverheid.nl/mensenonderzoek](http://www.rijksoverheid.nl/mensenonderzoek).

**1. General information**

The Radboud University Medical Center in Nijmegen initiated this study in collaboration with, among others, Isala Hospital in Zwolle, St Antonius Hospital in Nieuwegein, Amsterdam UMC in Amsterdam, Andros Clinics, Bravis Hospital in Roosendaal, and the HIFU Clinic in Etten-Leur. Below, we will refer to this group of hospitals as the “Sponsor”. Researchers, including physicians, clinical technologists, or (research) nurses, conduct the study in the various hospitals mentioned above.

Participants in medical scientific research are often referred to as subjects.

In the Netherlands, approximately 356 subjects are expected to participate, half of whom (178 subjects) will be treated with focal therapy and the other half (178 subjects) will receive the standard treatment for prostate cancer.

The Medical Research Ethics Committee METC-Oost Nederland has approved this study.

**2. What is the purpose of the study?**

In this study, we compare the effectiveness and safety of focal therapy with the effectiveness and safety of the standard treatment for prostate cancer. These **standard treatments** are either prostate surgery (also called radical prostatectomy) or radiation treatment of the prostate (also called radiotherapy). In **focal therapy**, only the tumor and a surrounding safety margin are treated. Most of the healthy prostate tissue is therefore not treated. We expect this to result in fewer side effects such as incontinence and impotence compared with prostate surgery or radiation treatment, but we would like to investigate this further.

**3. What is the background of the study?**

For people with intermediate-risk prostate cancer, several standard treatment options currently exist. One of these is prostate surgery (radical prostatectomy). During this procedure, the entire prostate is surgically removed, which may result in side effects such as impotence and incontinence. The other option is radiation treatment (radiotherapy). In this treatment, the entire prostate is irradiated with radioactive radiation. Extensive information regarding the advantages and disadvantages of both methods can be found in the “Prostate Cancer” decision aid.

At this time, we are conducting research on a new type of treatment for prostate cancer. In patients with intermediate-risk prostate carcinoma, we are investigating whether we can treat the tumor in the prostate using focal therapy. This is a treatment in which only the tumor and a surrounding safety margin are treated instead of the entire prostate.

In this study, focal therapy may be performed in three different ways: TULSA (tumor destruction using ultrasound through the urethra), HIFU (tumor destruction using ultrasound through the rectum), or IRE (tumor destruction using electrical current through the skin).

**4. How will the study be conducted?**

***How long will the study last?***

If you participate in the study, it will last a total of 60 months.

A schematic overview can be found in Appendix C.

***Step 1: Are you eligible to participate?***

First, we want to determine whether you are eligible to participate. Your urologist will discuss this with physicians from other involved specialties, such as (interventional) radiologists and radiation oncologists. If you are eligible, your urologist and one of the involved researchers will inform you accordingly.

***Step 2: Before treatment***

If you are eligible for the study and have given consent to participate, we will schedule an appointment with your urologist together with a researcher. During this visit, you may ask additional questions about the study. We will also ask you to complete several questionnaires.

For this study, we create 2 groups:

• Group 1. People in this group will receive focal treatment using TULSA (tumor destruction using ultrasound through the urethra), HIFU (tumor destruction using ultrasound through the rectum), or IRE (tumor destruction using electrical current through the skin).

• Group 2. People in this group will receive the standard treatment for prostate cancer.

This consists of either prostate surgery, or radiation treatment. Together with your urologist, you will discuss which treatment is most suitable for you.

Assignment to one of the groups will be determined by randomization. After randomization, you will be treated within 6 weeks. If you are assigned to the focal treatment group and this is necessary, you will be referred to a nearby center where the treatment is performed.

The type of focal therapy depends on which type is offered in your region. It is not possible to choose the type of focal therapy yourself. Afterward, your own hospital will carry out the follow-up visits.

***Step 3: examinations and measurements***

A schematic overview of all examinations and measurements can be found in Appendix C.

The men in group 1 will undergo one of the following 3 treatments: TULSA, HIFU, or IRE.

Information regarding these treatments is described below. These treatments are not available at all centers, and it may occur that you are referred to another hospital for treatment (Radboudumc, Isala, St Antonius, Amsterdam UMC, Andros Clinics, or the HIFU Clinic).

You will receive the treatment available in your region. The follow-up care will take place at your own hospital.

- TULSA

The TULSA treatment (tumor destruction using ultrasound through the urethra) is performed under general anesthesia while you are lying inside the MRI scanner. To prevent infections, you will receive antibiotics.

The TULSA device is inserted into the urethra. A balloon is inserted into the rectum in order to cool and protect it from damage during treatment. Using the MRI scanner, your prostate and the tumor inside it will be visualized again. On this MRI scan, the tumor and a surrounding safety margin are outlined. This area will be heated using ultrasound generated by the TULSA device. The heat destroys the tissue. During the heating process, new MRI images are continuously obtained, showing the temperature of the tissue. This allows the treatment to be performed very precisely and accurately. Immediately afterward, a new MRI scan with contrast fluid will be performed to assess the treatment effect.

The treatment will take approximately 2–3 hours in total. After the treatment, you will receive a catheter, which will be removed after a maximum of one week. You will remain in the hospital for one night for observation.

- HIFU

The HIFU treatment (tumor destruction using ultrasound through the rectum) is performed under spinal anesthesia and sedation. To prevent infections, you will receive antibiotics. The HIFU device is inserted into the rectum. The MRI scan performed before treatment will be used in combination with ultrasound images obtained during the procedure. This allows the ultrasound beam to be directed very precisely at a focal point within the tumor. This creates localized heat that destroys the tissue. In this way, the entire tumor and a surrounding safety margin are destroyed. The procedure will take approximately 2 hours. During the treatment, you will receive a catheter, which will be removed after one week. If everything goes well, you may return home the same day.

- IRE

The IRE treatment (tumor destruction using electrical current through the skin) is performed under general anesthesia. To prevent infections, you will receive antibiotics. You will receive a catheter to empty the bladder during the procedure. The MRI scan obtained before treatment is used during the procedure to determine the exact location of the tumor. Needles are inserted through the skin between the anus and the scrotum into the prostate surrounding the tumor to define the treatment area. Electrical current is conducted through the needles to destroy the prostate tumor. The procedure will take approximately 45 minutes. The catheter will be removed after one to two weeks. You will remain in the hospital for one night for observation.

Additional information brochures regarding focal treatments are available.

If you are treated with focal therapy, representatives of the company behind the focal therapy may observe the treatment during the procedure. They will not actively participate in the treatment or receive any of your personal data. They may observe the images created during the procedure, may support the treating physician if necessary, and ensure that the equipment is used according to protocol. The representative will not perform any medical actions involving you as a patient and will never be present at the treatment location in the hospital without a member of the treatment team present.

The men in group 2 will undergo either prostate surgery or radiation treatment of the prostate.

Information regarding these treatments can be found on the following websites:

[Prostaatverwijdering - Radboudumc](https://www.radboudumc.nl/patientenzorg/behandelingen/prostaatverwijdering) (Prostate removal)

[Bestraling bij prostaatkanker - Radboudumc](https://www.radboudumc.nl/patientenzorg/behandelingen/bestraling-bij-prostaatkanker) (Radiation therapy for prostate cancer)

In the decision aid of the Prostate Cancer Foundation, you can find additional information regarding these two standard treatment options: prostate surgery (radical prostatectomy) and radiation therapy (radiotherapy).

**Step 4: Follow-up**

For all patients, follow-up consists of 9 hospital visits: at 6 weeks, 3, 6, 9, 12, 24, 36, 48, and 60 months after treatment. During these visits, a small amount of blood will be drawn to check the PSA level in your blood. This is no different from standard care. We will also ask you to complete several questionnaires before or during each visit. This will take approximately 5–10 minutes each time. Additionally, at 18 and 30 months you will also receive a PSA check combined with an outpatient clinic appointment. During these visits, no questionnaires will be administered. Patients undergoing focal treatment will be asked to undergo an additional MRI scan of the prostate at 12, 24, 36, 48, and 60 months after treatment to assess whether the focal treatment was successful. This scan takes approximately 30 minutes.

In addition, 12 months after focal treatment, prostate biopsies will be taken from these patients to determine whether the focal treatment was successful. This procedure takes approximately 30–60 minutes.

After this, active participation in the study ends and you will continue to be monitored according to the standard follow-up protocol after prostate cancer treatment. In the background, we will continue to collect information about you up to 120 months after treatment (PSA values, possible MRI results, and/or biopsy results). The complete study schedule can be found in Appendix C.

**5. What agreements do we make with you?**

We would like the study to proceed properly. Therefore, we make the following agreements with you:

- During this study, you will not participate in any other medical scientific study.
- You will attend every appointment.
- You will contact the researcher in the following situations:
  - You want to start using other medications, including homeopathic remedies, herbal medicines, vitamins, or over-the-counter medications
  - You are admitted to or treated in a hospital
  - You suddenly experience health problems
  - You no longer wish to participate in the study
  - Your telephone number, address, or email address changes.
- Additionally, we advise you to follow the rules below during the first 6 weeks after treatment:
- You should not strain during defecation
- You should not have sexual intercourse
- You should not ride a bicycle
- You should not perform heavy physical labor
- You should consume alcohol only in moderation

**6. What side effects, adverse effects, or inconveniences may you experience?**

As with any treatment, complications may occur after **focal prostate treatment**, such as a (wound) infection or bleeding.

Important: **contact the hospital immediately** if:

- You have a fever above 38.5°C (101.3°F), or a temperature above 38°C (100.4°F) lasting longer than 24 hours
- You have severe (abdominal) pain that does not disappear after taking the prescribed pain medication or the maximum dose of paracetamol (4 times daily, 2 tablets of 500 mg)
- You suddenly become unable to urinate
- You experience severe bleeding and pass large blood clots in your urine
- You feel that you are losing urine through the anus.

During office hours, you may contact:

Urology Outpatient Clinic

After 5:00 PM and during weekends, contact:

The on-call urologist

If necessary, you may then report to the Emergency Department for treatment.

The following side effects occur frequently:

- Blood loss through urine, stool, and/or semen. To flush the bladder properly, we advise you to drink plenty of fluids (1.5–2 liters per day).

The following side effects sometimes occur:

- Urinary tract infection. This must be treated with antibiotics.
- Inability to urinate (urinary retention). This is usually temporary (approximately one week) and is caused by swelling of the prostate following treatment. You will then temporarily receive a catheter to empty the bladder.
- Unintentional urine leakage (incontinence). This is usually temporary because the muscles surrounding the bladder and urethra must adapt to the new situation. This may last several weeks to months.
- You may no longer ejaculate semen. Because part of the prostate is treated during the procedure, it is possible that you will no longer have ejaculation during orgasm. The sensation of orgasm itself usually does not change.
- Erectile dysfunction or impotence. The risk of this is very small because only a small part of the prostate is heated during focal treatment, and the temperature of surrounding structures, such as nerves, is carefully monitored.

The following side effects are rare but may be serious:

- A bloodstream infection (sepsis) may occur. To treat this, antibiotics must be administered through an IV, and you may need to remain hospitalized for several days.
- An abscess may develop in the prostate. This is a cavity filled with pus and is almost always caused by a prostate infection. To treat this, a drainage tube is inserted into the abscess so the pus can drain. Additional antibiotics are often administered as well.
- In very exceptional cases, an opening may develop between the rectum and the urethra after focal therapy. This is called a fistula and is a serious but rare complication that requires surgical treatment.

Damage to structures located near your prostate, such as the urethra, rectum, or bladder, cannot be completely excluded.

**Standard treatment**

Prostate surgery (radical prostatectomy) or radiation treatment (radiotherapy) of the prostate may also cause side effects. The main side effects after prostate surgery are: inability to control urination (incontinence), inability to achieve or maintain a proper erection (impotence).

After radiation treatment of the prostate, urinary symptoms (such as difficulty urinating, frequent urination (including at night), or difficulty holding urine), irritation of the rectum and anus (blood in stool), and erectile problems are common.

In the Prostate Cancer Foundation decision aid, you can find additional information regarding the risks and effectiveness of the two standard treatment options: prostate surgery (radical prostatectomy) and radiation therapy (radiotherapy).

**7. What are the advantages and disadvantages of participating in the study?**

Participating in the study may have advantages and disadvantages. We have listed them below. Please think carefully about them and discuss them with others.

If you participate in this study and receive focal therapy, we expect that you may experience fewer side effects such as incontinence and impotence, which can occur after prostate surgery (radical prostatectomy) or radiation treatment (radiotherapy). This could be an advantage for you, but it has not yet been scientifically proven.

Participating in the study may have the following disadvantages:

- You may experience side effects or adverse effects from the focal treatment, as described in Section 6.
- You may experience inconvenience from the examinations performed during the study. For example, blood collection may cause some pain or bruising.
- You must comply with the agreements associated with participation in the study.
- Additional time investment for completing questionnaires in both study groups.
- In the focal therapy group: additional time investment for the extra MRI scans at 12, 24, 36, 48, and 60 months after treatment, and discomfort/pain from the prostate biopsies after 12 months.

Another disadvantage of focal treatment is that only the tumor is treated and not the entire prostate. As a result, it is possible that the tumor is not completely treated or that a new tumor develops in the prostate. Other therapies, such as prostate surgery (radical prostatectomy) or radiation treatment, remain possible after focal therapy.

It is possible that something may accidentally be discovered during the study or during a follow-up MRI scan that is not directly relevant to the study but may be important for your health or that of your family members. In that case, your general practitioner or specialist will discuss with you what further action should be taken. The costs of this fall under your own health insurance.

*Do you not want to participate?*

You decide for yourself whether you participate in the study. If you choose not to participate, you will receive the standard treatment for prostate cancer. Your treating urologist can tell you more about the available treatment options and their advantages and disadvantages.

**8. When does the study stop?**

The researcher will inform you if new information becomes available about the study that is important for you. The researcher will then ask whether you still wish to continue participating.

The study will stop for you in the following situations:

- All examinations according to the schedule have been completed.
- The entire study has ended.
- You decide to stop participating yourself. You may stop at any time. Please inform the researcher immediately. You do not need to explain why you are stopping. You will then receive the standard treatment for prostate cancer again. For your safety, the researcher may arrange one or more follow-up examinations.
- The researcher believes it is better for you to stop. The researcher will still invite you for a follow-up visit.
- One of the following authorities decides that the study must stop:
  - Radboudumc or one of the other participating hospitals,
  - the government, or
  - the medical ethics committee that reviews the study.

*What happens if you stop participating in the study?*

The researchers will continue to use the data and body material (prostate tissue) collected up to the moment you stop participating.

The entire study ends once all participants have completed the study.

**9. What happens after the study?**

After the study, you will continue to be monitored by the urologist according to the standard protocol following prostate cancer treatment. This means that your PSA level will be checked regularly and, if clinically indicated, you may undergo scans and/or biopsies of your prostate.

We will continue to collect this information for at least 60 months after treatment.

*Will you receive the results of the study?*

Approximately 2 years after your participation, the researcher will inform you of the first results of the study.

**10. What will we do with your data and body material?**

If you participate in the study, you also give permission for us to collect, use, and store your data and body material.

*What data will we store?*

We will store the following data:

- your name
- your sex
- your address
- your date of birth
- information about your health
- (medical) data collected during the study
- medical images collected during the study
- prostate tissue collected after biopsies and/or prostate surgery

*What body material will we store?*

We will collect, use, and store prostate tissue collected after biopsies and/or prostate surgery.

*Why do we collect, use, and store your data and body material?*

We collect, use, and store your data, medical images, and body material in order to answer the research questions of this study and to publish the results.

*How do we protect your privacy?*

To protect your privacy, we assign a code to your data, medical images, and body material.

Only this code will be placed on all your data, medical images, and body material. The key to this code will be stored in a secure location within the hospital. Whenever we process your data, medical images, and body material, we will only use this code. In reports and publications about the study, no one will be able to identify that the information concerns you.

*Who can view your data?*

Certain individuals may still be able to view your name and other personal data without the code. This may include data specifically collected for this study, as well as information from your medical record.

These individuals are responsible for monitoring whether the researchers conduct the study properly and reliably. The following persons may have access to your data:

- Members of the committee monitoring the safety of the study.
- An auditor hired by the [researcher/sponsor] OR an auditor working for the [researcher/sponsor].
- National and international regulatory authorities.

These individuals are required to keep your data confidential. We ask for your permission to allow these persons access to your data. The Health and Youth Care Inspectorate may review your data without your permission.

*How long will we store your data and body material?*

We will store your data, medical images, and body material in the hospital for 15 years.

*May we use your data and body material for other research?*

Your data, medical images, and your (remaining) body material may also be important for other scientific research concerning prostate cancer or the improvement of (focal) treatment. For this purpose, your data, medical images, and body material will be stored in the hospital for 15 years. In the consent form, you indicate whether you agree to this. If you do not give permission, you may still participate in this study and receive the same care.

*What happens in the case of unexpected findings?*

During the study, we may accidentally discover something that is not directly relevant to the study but may be important for your health. The researcher will then contact your general practitioner or specialist. You will discuss with your doctor or specialist what further action should be taken. The costs of this fall under your own health insurance. By signing the form, you give permission to inform your general practitioner or specialist.

*Can you withdraw your consent for the use of your data?*

You may withdraw your consent for the use of your data at any time. You should inform the researcher if you wish to do so. This applies both to the use of your data in this study and to its use in other research. Please note: if you withdraw your consent after researchers have already collected data for a study, they may still use the data already collected.

*Would you like more information about your privacy?*

- Would you like to know more about your rights regarding the processing of personal data? Please visit [www.autoriteitpersoonsgegevens.nl](http://www.autoriteitpersoonsgegevens.nl).
- Do you have questions about your rights? Or do you have a complaint regarding the processing of your personal data? Please contact the person responsible for processing your personal data. For this study, this is:
  - Data Protection Officer, Radboudumc. See Appendix A for contact details and website.
- If you have complaints regarding the processing of your personal data, we recommend first discussing them with the research team. You may also contact the Data Protection Officer of Radboudumc or submit a complaint to the Dutch Data Protection Authority.

*Where can you find more information about the study?*

More information about this study can be found on the website [www.ClinicalTrials.gov](http://www.ClinicalTrials.gov). After completion of the study, the website may display a summary of the study results. You can find the study by searching for: “Effectiveness of focal therapy in men with prostate cancer (ENFORCE)”.

**11. Will you receive compensation for participating in the study?**

The study materials, additional tests, and treatments related to the study will not cost you anything. Your participation is greatly appreciated but entirely voluntary. No financial compensation will be provided for participation. For examinations and/or hospital visits that are additional to the standard treatment, you may receive travel reimbursement up to a maximum of €0.19 per kilometer.

**12. Are you insured during the study?**

Insurance has been arranged for everyone participating in this study. The insurance covers damage caused by the study, but not all types of damage. **Appendix B** contains more information regarding the insurance and its exceptions. It also explains to whom you can report any damage.

**13. We will inform your general practitioner and/or treating specialist**

The researcher will send a letter/email to your general practitioner and/or treating specialist informing them that you are participating in the study. This is for your own safety. In the event of side effects during participation, we may contact your doctor, for example regarding your medical history or the medications you are using.

**14. Do you have questions?**

You may ask the researcher or the research team any questions regarding the study. Would you like advice from someone who has no interest in the study? Please contact the independent expert, Dr. Frank Martens. See Appendix A for contact details. He is knowledgeable about the study but is not involved in conducting it.

Do you have a complaint? Please discuss it with the researcher or the physician treating you.

Would you prefer not to do this? Then contact the complaints officer/complaints committee of your hospital. Appendix A explains where you can find them.

**15. How do you provide consent for the study?**

You may first take time to think carefully about this study. Afterward, you will inform the researcher whether you understand the information and whether or not you wish to participate. If you wish to participate, you must complete the consent form included with this information letter. Both you and the researcher will receive a signed copy of this consent form.

Thank you for your time.

**16. Appendices to this information**

A. Contact details
B. Information regarding insurance
C. Schedule of study procedures

**Appendix A: Contact Details for Radboudumc**

**Researchers:**

- **Interventional Radiologist** (Principal Investigator)
  Department of Radiology
- **Urologist**
  Department of Urology

**Independent expert:**

- **Urologist**
  Department of Urology

**Complaints:**

- **Radboudumc Complaints Mediation Office**

Please note: not for medical complaints/side effects — for those, contact the researcher.

Radboudumc, Attn: Complaints Mediation

Complaints Mediation website: <https://www.radboudumc.nl/patientenzorg/uw-afspraak/meer-informatie/klachten>

**For more information about your rights:**

- **Data Protection Officer of the institution:**

Radboudumc, Attn: Data Protection Officer

Privacy website: <https://www.radboudumc.nl/patientenzorg/rechten-en-plichten/privacy>

**Appendix B: Information Regarding Insurance**

Radboudumc has arranged insurance for everyone participating in the study. The insurance covers damage that you suffer as a result of participating in the study. This concerns damage occurring during the study or within 4 years after the end of your participation in the study.

You must report the damage to the insurer within 4 years.

If you suffer damage due to the study, please report this to the following insurer:

The study insurer is:

Name of insurer: Centramed B.A.

Adres:

Telephone number:

E-mail:

Policy number:

The insurance provides coverage up to €650,000 per person and €5,000,000 for the entire study (€7,500,000 per year for all studies conducted by the same sponsor).

Please note: the insurance does **not** cover the following damage:

- Damage caused by a risk about which we informed you in this letter. However, this does not apply if the risk turned out to be greater than expected beforehand, or if the risk was highly unlikely.
- Damage to your health that would also have occurred if you had not participated in the study.
- Damage resulting from your failure to properly follow instructions or directions.
- Damage to the health of your children or grandchildren.
- Damage caused by an existing treatment method, or by research into an existing treatment method.

These provisions are stated in the “Mandatory Insurance Decree for Medical Scientific Research Involving Human Subjects 2015.” This decree can be found in the Dutch government legislation database (<https://wetten.overheid.nl>).

**Appendix C: Schedule of study procedures**

Below you will find an overview schedule of the study. For comparison, we have placed the treatment and follow-up schedules of the standard treatments for prostate surgery (radical prostatectomy) and radiation therapy (radiotherapy) alongside.

Procedures that are additional within the context of this study are marked with an *.

In the decision aid of the Prostate Cancer Foundation, you can find more information about the risks and effectiveness of the two standard treatment options: prostate surgery and radiation therapy.

| **Treatment schedule** | **Focal treatment**  (IRE, TULSA, HIFU) | **Prostate surgery** (Radical prostatectomy) | **Radiation therapy**  (Radiotherapy) |
| --- | --- | --- | --- |
| **Voor de behandeling** | - - Visit to anesthesiologist   - PSA   - Questionnaires* | - - Visit to anesthesiologist   - PSA   - Questionnaires* | - - CT – and MRI scan   - (Placement of gold markrs)   - PSA   - Questionnaires* |
| **Behandeling** | - - Focal treatment under general or spinal anesthesia. Discharge the same day or after 1 day. | - - Prostate surgery under general anesthesia, followed by approximately 2 days in hospital. | - - 5–35 radiation sessions over 2.5–7 weeks. |
| **6 weken**  **3, 6, 9 maanden** | - - PSA   - Questions about side effects and post-treatment course   - Questionnaires* | | |
| **12 maanden** | - - PSA   - Questions about side effects and post-treatment course   - Questionnaires*   - MRI*   - Prostate biopsy* | - - PSA   - Questions about side effects and post-operative course   - Questionnaires* | |
| **24, 36, 48, 60 maanden** | - - PSA   - Questions about side effects and post-treatment course   - Questionnaires*   - MRI*   - Prostate biopsy if MRI suggests recurrence* | - - PSA   - Questions about side effects and post-treatment course   - Questionnaires* | |
