# Supplementary material for: (Cost-)effectiveness of focal therapy versus radical therapy (standard of care) in the treatment of men with intermediate-risk prostate cancer: study protocol for the ENFORCE focal randomised controlled trial
Source: BMJ Open. 2026 Jul 8;16(7):e117276. doi: 10.1136/bmjopen-2026-117276 (PMC13347909; doi:10.1136/bmjopen-2026-117276)
Supplement: online supplemental file 2 [file bmjopen-16-7-s002.docx]

*Disclaimer: this document is an English translation of the original Dutch informed consent form for publication purposes. Only the Dutch version was approved by the Medical Ethics Review Committee and used during participant enrollment.*

**Informed consent form for participant**

Belonging to
**Effectiveness of focal therapy in men with prostate cancer (ENFORCE)**

- I have read the information letter. I was also able to ask questions. My questions were sufficiently answered. I had enough time to decide whether I want to participate.
- I understand that participation is voluntary. I also understand that I can decide at any time not to participate in the study or to stop participating. I do not have to give a reason for stopping.
- I give the researcher permission to inform my general practitioner/specialist(s) who are treating me that I am participating in this study.
- I give the researcher permission to request information from my general practitioner/specialist(s) who are treating me regarding my medical history and medication use.
- I give the researcher permission to provide my general practitioner or specialist with information about unexpected findings from the study that are relevant to my health.
- I give the researchers permission to collect and use my data and/or body material. The researchers will use this only to answer the research question of this study.
- I understand that, for monitoring of the study, some people may review all my data. These people are listed in the information letter. I give permission for these people to access my data for this monitoring.
- Please tick yes or no in the table below:

| I give permission for my data to be stored and used for other research, as described in the information letter. | Yes ☐ | No ☐ |
| --- | --- | --- |
| I give permission for my (remaining) body material to be stored and used for other research, as described in the information letter. The material will be stored for up to 15 years for this purpose. | Yes ☐ | No ☐ |
| I give permission to be approached after this study to participate in a follow-up study. | Yes ☐ | No ☐ |
| I give permission that, in case I die during the study period, my official cause of death may be requested from Statistics Netherlands (CBS). | Yes ☐ | No ☐ |

- I agree to participate in this study.

My name is (participant): ………………………………..
Signature: ……………………… Date: __ / __ / __

-----------------------------------------------------------------------------------------------------------

I declare that I have fully informed this participant about the above-mentioned study.

If information becomes available during the study that may influence the participant’s consent, I will inform the participant in a timely manner.

Name of researcher (or representative):……………………………….
Signature:……………………… Date: __ / __ / __

-----------------------------------------------------------------------------------------------------------

<if applicable>

Additional information provided by:

Name:………………………………..

Role:………………………………

Signature:……………………… Date: __ / __ / __

-----------------------------------------------------------------------------------------------------------

*The participant will receive a complete information letter, together with a signed copy of the consent form.*
