# Supplementary material for: (Cost-)effectiveness of focal therapy versus radical therapy (standard of care) in the treatment of men with intermediate-risk prostate cancer: study protocol for the ENFORCE focal randomised controlled trial
Source: BMJ Open. 2026 Jul 8;16(7):e117276. doi: 10.1136/bmjopen-2026-117276 (PMC13347909; doi:10.1136/bmjopen-2026-117276)
Supplement: Supplementary file 3 [file bmjopen-2026-117276.reviewer_comments.pdf]

PEER REVIEW HISTORY

BMJ Open publishes all reviews undertaken for accepted manuscripts. Reviewers are asked to complete a checklist review form and are provided with free text boxes to elaborate on their assessment. These free text comments are reproduced below.

ARTICLE DETAILS

Title (Provisional)

(Cost-)effectiveness of focal therapy versus radical therapy (standard of care) in the treatment of men with intermediate-risk prostate cancer: study protocol for the ENFORCE focal randomized controlled trial

Authors

te Molder, Lauren P.W.; Sedelaar, Michiel; Bomers, Joyce G R; Boomsma, Martijn F; De Haan, Tjard D; Van Melick, Harm H; Meijerink, Martijn R.; Beerlage, Harrie P; Van Aubel, Olav; Collette, Eelco R; Grutters, Janneke; Hannink, Gerjon; Rovers, Maroeska M; Fütterer, Jurgen J; ENFORCE Focal consortium, on behalf of

---

VERSION 1 - REVIEW

**Reviewer**	**1**
**Name**	**Mangar, Stephen A.**
**Affiliation**	**Imperial College London**
**Date**	**04-Mar-2026**
**COI**	**None**

The authors are to be congratulated on their manuscript submission.

It is well written, with a clear methodology and is concise.

The study addresses an important unmet clinical need in treating men with intermediate risk prostate cancer aiming to achieve a cure whilst minimising toxicity.

It is an ambitious study ad one where the authors do acknowledge the challenges and limitations.

Issues that the authors need to consider:

-Justification for choosing the 36-month time interval for clinical effectiveness of the intervention. Bearing in mind that the cohort is highly selected for intermediate risk disease, the authors may find that there are not enough events of recurrence to make any meaningful conclusions, especially with regards to clinical effectiveness.

-I think it is important for the authors to state whether androgen deprivation therapy will be used with radiotherapy treatment and for how long. How do the authors think this may influence outcome and quality of life data?

-The authors also need to make it clear what the dose and fractionation of radiotherapy would be permissible, as if there is variation this could skew the QOL data and overall outcome.

-Is a nerve sparing prostatectomy being used / or stated as this will impact quality of life collection?

-With regards to those receiving focal therapy – I note that an MRI and biopsy will be performed routinely at 12 months, but then only performed on indication thereafter. I would recommend the authors being very clear as to what the indications would be – i.e. serial PSA measurements.

-with regards to inclusion criteria- are these patients going to be fully staged – i.e. MRI, CT and bone scan for the purposes of this study, and if not a justification as to why not?

VERSION 1 - AUTHOR RESPONSE

Reviewer 1:

1. Justification for choosing the 36-month time interval for clinical effectiveness of the intervention. Bearing in mind that the cohort is highly selected for intermediate risk disease, the authors may find that there are not enough events of recurrence to make any meaningful conclusions, especially with regards to clinical effectiveness.

We acknowledge the limitations of a 3c-month follow-up period. However, we believe this timeframe is appropriate, as treatment effectiveness in prostate cancer is reflected by early biochemical and clinical failure events, which are validated surrogates for metastasis-free survival. Detecting differences in overall survival would require an impractically long follow-up period, given the high baseline survival and the presence of competing mortality risk.
Nonetheless, participants will be followed for up to 5 years, allowing the collection of additional outcome data beyond the primary analysis.

I think it is important for the authors to state whether androgen deprivation therapy will be used with radiotherapy treatment and for how long. How do the authors think this may influence outcome and quality of life data?

We share the opinion of the reviewer that this subject was not addressed in our first manuscript. We clarified the standard of care performed regarding the use of androgen deprivation therapy (ADT) in patients with intermediate-risk prostate cancer. Pages 11-12, lines 250-252. "According to the current national guidelines, the addition of androgen deprivation therapy (ADT) is not yet recommended for patients with intermediate-risk PCa with PSA level of 20ng/mL or lower."
Although ADT is an established component of prostate cancer treatment, our national guidelines, which are followed in this study, do not recommend ADT for patients with intermediate risk prostate cancer receiving radiotherapy with a PSA level of 20ng/mL or lower. European Guidelines reference studies such as Krauss et al. (2023) and the STAMPEDE trial, however, these studies either do not provide conclusive evidence for the use of ADT in intermediate-risk prostate cancer or include populations that differ from our trial cohort. As within this study, we aim not to interfere with standard

clinical care, we do not expect ADT to be added for patients receiving radiotherapy. However, if ADT were administered, it could influence biochemical recurrence and metastasis-free survival, although no improvement in overall survival is expected based on current evidence. ADT may both have positive effects on prostate cancer-related quality of life (e.g. urinary symptoms) and negative effects due to side effects in the hormonal changes, including mood alterations, sexual dysfunction, weight gain and other ADT-associated symptoms.

2. The authors also need to make it clear what the dose and fractionation of radiotherapy would be permissible, as if there is variation this could skew the QOL data and overall outcome.

Thank you for this suggestion. The manuscript has been updated with information regarding the dose and fractionation used in radiotherapy in this study. Page 11-12, lines 24c-250. "Patients receiving RT, will be treated as per national guidelines. Therapies administered, can include RT with hypofractionation (28 fractions with 2.5Gy, total dose of 70Gy, or, 20 fractions with 3.1 Gy, total dose of c2Gy) and RT with ultrahypofractionation (5 fractions with 7.25Gy, total dose of 3c.25Gy). All RT regimens provide bio-equivalent doses similar to conventional RT consisting of 35 fractions with 2Gy."

While differences in radiotherapy modalities have the potential to affect quality of life (QoL) outcomes, available data suggests that these variations do not lead to long-term QoL differences. This may be explained by the bio-equivalent dose of the various treatment regimes, all of which correspond to conventional radiotherapy delivered in 35 fractions of 2Gy, resulting in a total dose of 70-80Gy.

3. Is a nerve sparing prostatectomy being used / or stated as this will impact quality of life collection?

As suggested by the reviewer, this is now clarified in the manuscript. Page 11, lines 244-24c. "For RP, different types of approaches are possible including nerve-sparing and non-nerve sparing options, depending on tumor characteristics and anatomical considerations."
In line with standard of care, all types of (robot-assisted) prostatectomy may be performed, including nerve-sparing, NeuroSAFE, and non-nerve-sparing approaches. The surgical technique is selected based on tumor characteristics and anatomical considerations.

We do not expect these variations to substantially impact study outcomes, as treatment approaches are anticipated to be evenly distributed. The same principles apply to focal therapy, where the primary goal is oncological control, with nerve preservation attempted when oncologically feasible.

4. With regards to those receiving focal therapy – I note that an MRI and biopsy will be performed routinely at 12 months, but then only performed on indication thereafter. I would recommend the authors being very clear as to what the indications would be – i.e. serial PSA measurements.

As requested, we have updated the manuscript to clarify this subject. Page c, lines 144-147. "For the focal therapy arm, at 12 months an MRI as well as prostate biopsies will be performed. In the years thereafter, a prostate MRI will be performed yearly with additional biopsies on indication, such as rising PSA levels over time, suspicious MR findings in the treated zone, or newly identified lesions outside the treated area."
Following focal therapy, patients will undergo annual mpMRI for up to 5 years. At 12 months, all patients with focal therapy will receive protocol-mandated biopsies. Thereafter, additional biopsies may be performed in response to clinical triggers, including rising PSA levels, suspicious MRI findings such as early enhancement or diffusion restriction in the treated area, or newly identified lesions outside the

treated area.

5. With regards to inclusion criteria- are these patients going to be fully staged – i.e. MRI, CT and bone scan for the purposes of this study, and if not a justification as to why not? *Thank you for this question. The paragraph regarding patient characteristics in the manuscript is updated. Page S, lines 221-224.”Patients will be included after being diagnosed with intermediate-risk prostate cancer and having completed the diagnostic work-up according to national guidelines. At a minimum, all patients will have undergone clinical examination, PSA testing, prostate MRI, and prostate biopsies. Additional staging procedures are not part of standard staging for this risk category in the Netherlands.”.*

Routine PSMA-PET, CT or bone scans are not part of standard staging for this risk category in the Netherlands. PSMA-PET-CT may be performed when clinically indicated, for example, in the presence of an unfavorable intermediate-risk Gleason score.
